# TeloTool: a new tool for telomere length measurement from terminal restriction fragment analysis with improved probe intensity correction

**DOI:** 10.1093/nar/gkt1315

**Published:** 2013-12-22

**Authors:** Janett Göhring, Nick Fulcher, Jaroslaw Jacak, Karel Riha

**Affiliations:** ^1^Max F. Perutz Laboratories, Medical University of Vienna, Vienna 1030, Austria, ^2^Gregor Mendel Institute, Vienna 1030, Austria, ^3^Institute for Applied Physics, Johannes Kepler University Linz, Linz 4040, Austria and ^4^Upper Austria University of Applied Sciences, Campus Linz, Linz 4020, Austria

## Abstract

Telomeres comprise the protective caps of natural chromosome ends and function in the suppression of DNA damage signaling and cellular senescence. Therefore, techniques used to determine telomere length are important in a number of studies, ranging from those investigating telomeric structure to effects on human disease. Terminal restriction fragment (TRF) analysis has for a long time shown to be one of the most accurate methods for quantification of absolute telomere length and range from a number of species. As this technique centers on standard Southern blotting, telomeric DNA is observed on resulting autoradiograms as a heterogeneous smear. Methods to accurately determine telomere length from telomeric smears have proven problematic, and no reliable technique has been suggested to obtain mean telomere length values. Here, we present TeloTool, a new program allowing thorough statistical analysis of TRF data. Using this new method, a number of methodical biases are removed from previously stated techniques, including assumptions based on probe intensity corrections. This program provides a standardized mean for quick and reliable extraction of quantitative data from TRF autoradiograms; its wide application will allow accurate comparison between datasets generated in different laboratories.

## INTRODUCTION

Telomeres represent nucleoprotein structures that cap the natural ends of linear eukaryotic chromosomes. Telomeric DNA consists of tandem repeat arrays of TTAGG-like sequences at terminal chromosome ends, e.g. T_2_AG_3_ in mammals, T_3_AG_3_ in *Arabidopsis* and TG_1__–__3_ in budding yeast. The mean length of telomeric DNA is thought to mainly derive from a homeostatic balance between attrition due to the end replication problem and elongation via the reverse transcriptase telomerase (1). However, telomerase is not active within human somatic cell populations and thus, telomere shortening serves as a cellular senescence marker limiting cell proliferation capacity. When cells have undergone a large number of cell divisions, telomeres shorten to critical levels where they elicit a strong DNA damage response that may ultimately lead to chromosome fusions and mass genomic instability. Ectopic activation of telomerase is considered to be an important mechanism in tumor etiology to overcome one of many tumor suppression barriers and to allow immortalization of somatic cells and cancer progression. Therefore, the study of telomere length within organisms is essential to understand the mechanistic aspects of telomere maintenance through analysis of mutants displaying telomeric defects (2), study of telomere length in respect to human disease and organismal aging (3) or to study variation of length in natural populations and its contribution to the definition of lifespan (4,5).

A number of assays currently exist to measure telomere length from a number of organisms and tissues. The use of certain techniques essentially depends on the nature of the study; some techniques focus on telomere length at specific telomeres or within single cells, while others can measure telomere length distribution over whole tissues or organisms. Terminal restriction fragment analysis (TRF) is a popular choice within the field; this technique requires digestion of genomic DNA with frequently cutting restriction enzymes that exclude the telomeric sequence, gel electrophoresis of digested DNA and Southern blotting using a telomeric probe [for detailed protocol see (6)]. Other techniques such as quantitative PCR (qPCR)-based methods, Q-fluorescence *in situ* hybridization (FISH)/Flow-FISH, single telomere elongation length analysis and primer extension telomere repeat amplification are also available (7–15); despite this, TRF-based measurements are still considered the gold standard within the field as they provide data on absolute telomere length and heterogeneity [for an extensive review on the advantages and disadvantages of each technique, refer to (16)].

Certain challenges arise in the analysis of TRF data including the extraction of telomere length information from Southern blots. Because telomere length is not uniform over an entire tissue or organism, the TRF signal is presented as a heterogeneous smear. Methods outlining how this type of data can be accurately analyzed to provide precise information on telomere length and heterogeneity are not well defined. In the past, only a few statistical approaches for TRF smear analysis have been described; previous methods, however, do not fully correct for methodical biases. In fact, these analysis methods can create additional biases due to the imprecise estimation of parameters and boundaries. One major example includes the calculation of mean telomere length by averaging probe intensity over the entire lane (17–21). This method can only be adopted if the origin of the distribution was tested beforehand; if the distribution does not follow a normal distribution, the mean cannot be used as a parameter to describe the telomere length distribution. Further biases are also introduced by erroneous probe intensity corrections. In the case of standard Southern blotting techniques where telomeric DNA is denatured, probes in saturation would randomly bind to all available telomeric repeats. This means that raw data need to be corrected for probe intensity because of the increased number of binding sites at longer telomeres (6,22). Probe intensity correction can be performed simply by dividing the raw data by the number of available binding sites (22). However, this approach leads to an underestimation of the actual mean telomere length, as probes bind in a probabilistic way (23). Despite the fact that TRF-based measurements are so commonly used in telomere biology, there is currently no standardized way to analyze TRF blots.

In this article, we outline current problems with the analysis of TRF data and present TeloTool, a freely available program to perform correct statistics on TRF smears. TeloTool takes all known problems and challenges with TRF analysis into account and provides a statistically correct method to standardize analysis of TRF data. We compare TeloTool with a previously published analysis tool for TRF smears called Telometric (22). Finally, we will discuss all known problems that affect TRF smear dynamics and suggest biological applications for TeloTool.

## MATERIALS AND METHODS

**Figure 1.**
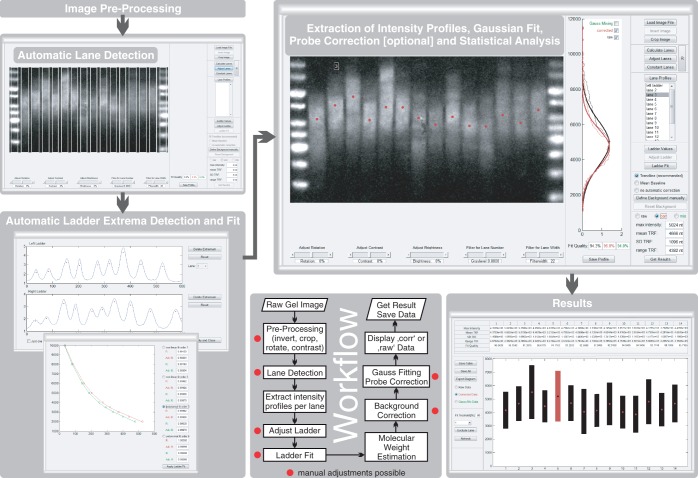
Typical work flow for TRF analysis with TeloTool. Raw TRF scans in 8- or 16-bit TIFF formats can be loaded directly into TeloTool and easily rotated and cropped for analysis. Lanes are then detected automatically, ‘Filter for lane number’ and ‘Filter for lane width’ sliders can be used to fine tune lane recognition. Marker bands are then detected and a fit is applied to determine the molecular weight over the gel image, incorrectly identified bands must be removed. A Gauss curve is fitted to each lane and data are automatically subject to probe intensity corrections. The user can choose to display the resulting data for the fitted raw data or the probe-corrected data. After analysis, data are presented in a graph displaying mean and range values along with exportable length values in Microsoft Excel format.

TeloTool was developed in Matlab (Mathworks) and runs on a 64 bit windows platform, which requires installation of the MATLAB Compiler Runtime [version 7.17 (R2012a), freely available at the Mathworks web page http://www.mathworks.com/products/compiler/mcr/]. TeloTool is available for download at (https://github.com/jagoehring/TeloTool; the source code is available at http://www.mathworks.com/matlabcentral/fileexchange/44573) along with specific details for installation and operation. Here, we describe the typical workflow for data analysis in TeloTool and describe features exhibited by the program; this workflow is illustrated in Figure 1.

### Image preprocessing

TeloTool provides a complete image analysis pipeline for the processing of raw TRF scans to extract telomere length data. In principle, any 8- or 16-bit TIFF file can be used for analysis, as long as the exposure time is set within the linear range of the detector (no saturation of the peak signals). TeloTool automatically converts RGB images to grayscale without information loss. As soon as the image has been loaded, the user may perform standard image manipulation steps such as contrast and brightness adjustments, cropping and image inversion. Contrast and brightness adjustments are for the convenience of the user, as they only transform the visible gel image; data analysis, however, is always performed using the original raw image.

TRF scans containing artifacts such as bubbles and blotches are always a concern during analysis; therefore, we recommend that the user judge the integrity of each gel as the accuracy inferred by TeloTool is, of course, dependent on the quality of the raw data. When the integrity of certain smears within a TRF is questionable, it is recommended to exclude these from analysis. When ‘smiley’ gels are a common problem, introduction of internal marker lanes during the gel preparation will allow the user to accommodate such effects.

### Lane detection

The next step in analysis requires recognition of the gel lanes, a process which is automated by TeloTool. This is based on the recognition of local minima within the x-projected intensity profile over the whole gel. It is possible to adjust the local minima detection by changing two parameters: (i) the slider ‘Filter for Lane Number’ adjusts the global image threshold using Otsu’s method (24). This threshold is used to convert an intensity image to a binary mask, which is 1 for each pixel with an intensity above the threshold. Changing the slider position influences the number of lanes that are recognized. If the ladder’s intensity is much higher than the samples, lane detection may be more time-consuming as the user has to find the correct threshold. (ii) After the logical mask has been generated, TeloTool x-projects the mask creating an ‘intensity’ profile of the gel. The slider ‘Filter for Lane Width’ influences the erosion of the local minima, which define the borders between the lanes. Changing the slider position basically widens or narrows the lane width. If the user is finally content with the detected lanes, the user may equalize their width by selecting ‘Constant Lanes’. It detects the narrowest lane and changes all others to be the same width. This may be important for comparison of intensities between lanes of the same gel. If there is any need for further adjustment of the lanes (e.g. in the presence of gel defects), the user may perform this manually with ‘Adjust Lanes’, which opens a new graphical user interface (GUI). Here, the user can change the boundaries of already detected lanes or add new lanes. It is important to note that the user can only manipulate the lane boundaries in x and not in y, i.e. the analysis window always contains the whole gel lane. This only can be varied by cropping the image, but the user is strongly advised to analyze the whole TRF smear. In some cases, smears from short interstitial centromeric sequences containing limited telomeric repeats can be observed at the bottom of the membrane, this can occur when using certain restriction enzymes. These smears are easily identifiable and should not be included in the analysis.

### Ladder manipulation and fit

After the lanes have been defined to the user’s satisfaction, the intensity profiles of the lanes are plotted in an extra analysis axes. TeloTool recognizes both first and last lanes as molecular markers and automatically detects bands within these lanes. At this point, the user then has to define the molecular weight values of the ladder and delete incorrectly recognized ladder extrema; ladder values are stored until manually changed. The analysis will be more accurate if the gel contains two flanking ladders so that it is possible to correct for a shift in migration throughout the gel. There is the option to define just one ladder within the ‘Adjust Ladder’ GUI; however, because gel shifts are a common phenomenon, it is recommended to use two ladders.

From the theory of DNA propagation in electrophoresis gels, it is well known that the DNA velocity displays a length dependency (25,26). Short sequences pass easier through the ‘pores’ of a gel than bulky large sequences, as they often contain secondary structures and, thus, migrate slower. To correct for non-linear DNA migration through gels, four different functional fits are available (Ladder Fit) to transform the pixel position of the gel into molecular weight. If no fit has been chosen, TeloTool automatically fits the ladder with a polynomial function of the third order (25), which is sufficiently accurate in most cases. However, for a better comparison, and to simplify the choice between the fits, the coefficient of determination (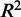) and the adjusted version (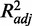) are calculated and displayed.

The residual sum of squares 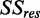 is defined as

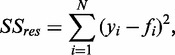

where the determined pixel position for each band of the ladder is 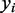, the associated modeled values are 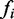 and 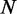 is the number of detected bands of the ladder.

The total sum of squares 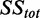 is defined as

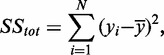

where 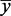 is the mean of the observed data.

The coefficient of determination 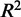 is defined as

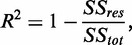

and the adjusted coefficient of determination 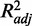 is defined as

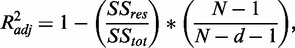

where 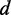 is the degrees of freedom of the polynomial.

### Statistical analysis

After establishing the correct size vector throughout the gel image, the intensity profile from every lane is automatically displayed according to its molecular weight. Eventually, probe intensity distributions over each smear can be assessed for mean telomere size and dispersion values. In detail, the data arising from our test sets can be described by a Gaussian curve, which has been found to be the best fitting function. The Gaussian probability density function is fitted into 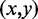 points using the iterative least mean square method that minimizes the residuals with each iteration (the maximum number of iterations is 50). The Gaussian fit is finally given by

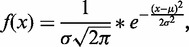

where 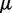 is the mean and 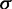 the standard deviation of the distribution.

Therefore, the fitted curve can be used to calculate the mean and standard deviation of the distribution resulting in an improved estimation of the mean and the range of the telomere length. To allow the user to judge how well the curve describes the original data, the fit quality is displayed beneath the axis containing the profile. The fit quality resembles the coefficient of determination 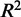 and is computed as described previously (see ‘ladder manipulation and fit’).

### Probe dynamics and correction

TRF analysis can be subdivided into two separate modes of probe hybridization: (i) in-gel hybridization where telomeric DNA is not denatured and the probe binds only to the G-overhang of the native telomere and (ii) standard Southern blotting techniques where the DNA is denatured after electrophoresis and transferred to a membrane where the probe can bind to the entire telomeric sequence. Traditionally, in the case of the second technique, it is assumed that probes bind randomly to their target sequence; because they are in saturation, all available target sites should be bound. Therefore, TRF smears from the in-gel protocol represent the true telomere length distribution, whereas the smears of denatured DNA need to be corrected for probe intensity before analysis (6,22). As previously mentioned, current probe correction techniques involve dividing probe intensity by the molecular weight that is assumed to be proportional to the number of all possible binding sites; this assumes all sites are saturated and every label is intact. Because probes likely bind in a probabilistic manner, it is statistically relevant to correct the raw data for probe intensity by mathematical modeling.

**Figure 2.**
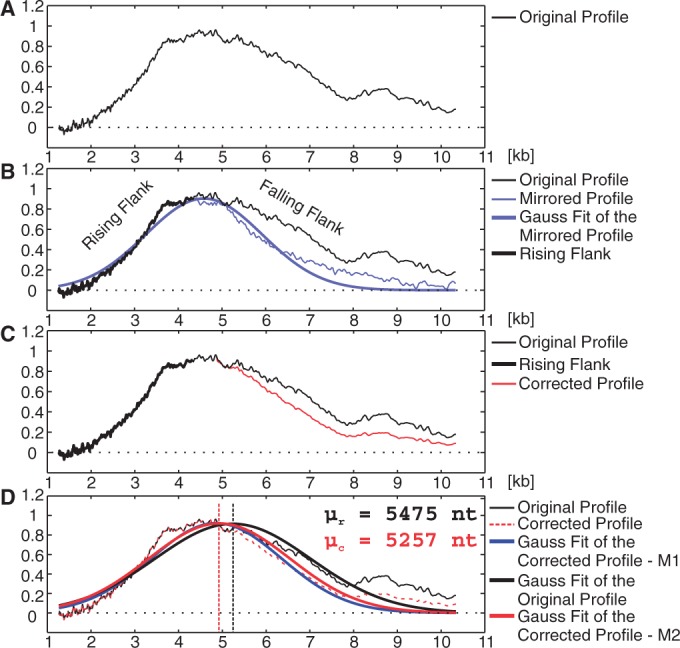
Optional probe correction by TeloTool. The intensity analysis of one lane usually results in an asymmetric profile (**A**). The falling flank (high molecular weight) is especially heterogeneous and prone to local maxima. The binding probability of the probe is nearly linear in the range of smaller telomeres, i.e. they contain the largest number of validly bound probes. Therefore, the rising flank of the data is used to mathematically model the intensity-corrected profile. First, the rising flank is extracted, mirrored on the *x*-axis (correction of profile symmetry based on the rising flank) and subsequently fitted with a first-order Gaussian function (**B**). The Gaussian fit is used for the correction of the falling flank of the intensity profile. TeloTool provides two different correction methods. (i) For each point of the falling flank, TeloTool calculates the mean between the fitted Gaussian function and the original data. (ii) TeloTool mixes two different Gaussian functions; one is obtained by fitting the original data and a second Gaussian is fitted into the mirrored left flank profile. Finally, the intensity-corrected profile (**C**) is fitted with another Gaussian function and the mean, sigma and fit quality of the fit is displayed in the result section (**D**). µ_r_ and µ_c_—mean telomere length for the corrected (c) and uncorrected (r) data set.

To explain the approach to mathematical modeling of TRF smear profiles and the correction for probe intensity, its application to raw data is illustrated in Figure 2. First, the raw signal profile (Figure 2A) is subject to a preprocessing step where the program extracts the low molecular weight flank (up to the maximum intensity) (25) and performs a mirror reflection along the *y*-axis (Figure 2B). Herein, we have to point out that for long repetitive sequences like telomeres, issues such as probe labeling stoichiometry (27), self-complementary metastable states of telomeric DNA (28,29), complex hybridization kinetics due to probe diffusion in porous supports (30–32) and diffusion of long telomeres in porous supports (33,34) play a crucial role in hybridization kinetics (see Supplementary Data S1 for theoretical considerations). Therefore, the profile correction can be reduced to the correction based on low molecular weight flank. The mirrored curve is fitted via a first order Gauss function. The same mirrored Gaussian is then used for fitting of the high molecular weight flank. Subsequently, the error between the new ‘falling’ flank and the original flank is calculated. If the error approaches a minimum, it is used to correct the original data creating the new ‘corrected’ data set (Figure 2C). This data set is again fitted with a Gaussian curve and finally, the fit quality is calculated (Figure 2D).

The program has two implemented methods for profile correction: Let 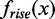 be the Gauss-fit function of the mirrored original data of low molecular weight flank (to the maximum intensity, called rising flank). The first correction method calculates the mean value between the mirrored Gauss-fitted distribution and the original data for each point of the high molecular weight flank (up to the maximum intensity, called falling flank).

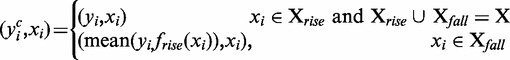



The second method uses the Gaussian fit 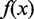 of the original data and the mirrored Gauss-fitted distribution 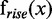 of the low molecular weight flank for the profile correction.

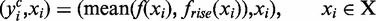

The new fitted distribution is derived by Gaussian mixing of the two distributions described earlier in the text.

The corrected points 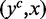 are finally approximated with the new Gaussian probability density function 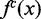. In most cases, results obtained from method 1 (Gaussian profile) and method 2 (Gaussian mixing) exhibit a similar fit quality. This additionally confirms the high similarity of the TRF smear profile to a Gaussian distribution.

### Background correction

Furthermore, the user may choose between three background subtraction options: (i) the ‘trendline’ option detects the minimum of the first and last 10 pixels of the lane and calculates a smooth line between these values along the lane and then the trendline is subtracted from the raw data. (ii) The ‘baseline’ option calculates the mean between these two values and subtracts the mean from the original data. (iii) The user may manually define an area of the gel as background. Subsequently, the area mean is subtracted from the raw data. All aforementioned options are based on background subtraction; we recommend usage of the ‘trendline’ option, as it subtracts an intensity gradient from the whole intensity profile. Background correction has not been implemented, as available methods cut off signal intensity especially from the typical smooth ‘rising’ flank of the TRF smear; this flank is needed for our mathematical modeling approach.

### Results summary

Finally, the resulting analysis of the gel is displayed in a new GUI. The mean, the standard deviation, the range, the max intensity and the fit quality are summarized within a table for the ‘raw’ data and the ‘corrected’ data. The user may export individual tables or all at once into the .xls format (Microsoft Excel). Furthermore, a simplified graph of the gel is created displaying bars that symbolize 1.18 times the calculated standard deviation (covers 75% of the data) and the mean. If the fit quality falls below a manually adjustable threshold, the bar is displayed in red and the user may exclude it from the graph. The graph, as well as the individual intensity profiles, can be exported as a .png, .eps, .pdf and .tif file. For the conversion to the vector format (eps and pdf), the user has to manually install respective open-source programs (Ghostscript at http://www.ghostscript.com and pdftops at http://www.foolabs.com/xpdf).

### Statistical analysis within the manuscript

The Kolmogorov-Smirnov test (KS-test) is used to compare two samples (two-sample KS-test), which tests whether the samples are drawn from the same distribution (the null hypothesis). The two-sample KS-test is one of the most useful and general non-parametric methods for comparing two samples, as it is sensitive to differences in both location and shape of the empirical cumulative distribution functions of the two samples. The KS-test can be modified to serve as a goodness of fit test. The null hypothesis is rejected if level α < 0.05.

## RESULTS

### Comparison TeloTool and Telometric

Telometric (22) is a previously published program for the purpose of TRF analysis; however, biases have been previously identified using this program (35). Telometric functions by averaging the intensity distribution over the entire lane of the gel; this then allows the program to calculate mean and median telomere length information. One major problem with this approach is that the distribution’s origin is not tested beforehand; therefore, it is assumed that each smear follows a normal distribution.

**Figure 3.**
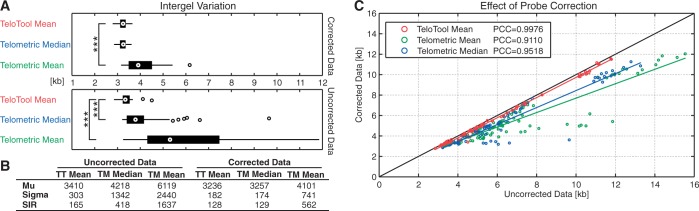
Comparison of intergel variance between TeloTool and Telometric and effects of probe correction. (**A**) To test the variation of the same sample between different TRF gels, 31 lanes containing the Col-0 accession have been analyzed by both programs. TeloTool’s mean intergel variation for corrected data does not differ from Telometric’s median intergel variation. However, there are vast differences between intergel variation for uncorrected data (KS-Test, two-sided, α at 0.05, ***P < 0.005). (**B**) Comparison of the statistical parameters of both programs (visual display of the data can be found in panel A). TM—Telometric; TT—TeloTool, mu—mean of all the mean telomere lengths, sigma—standard deviation and SIR—semi-interquartile range. (**C**) Effect of probe correction from both programs. The corrected and uncorrected data of five different accessions was plotted and the linear regression graph calculated. Telometric’s telomere length values, calculated from probe-corrected data, decrease in size in comparison with the uncorrected data. This suggests that the formula that calculates the probe correction in Telometric leads to a vast underestimation of telomere length at longer telomeres. The respective data can be found in Supplementary Table S1. PCC, Pearson’s correlation coefficient.

To compare TeloTool with Telometric, we analyzed TRF data from *Arabidopsis thaliana* to assess the intergel variation experienced with each program. We measured 33 different TRF smears of the Col-0 accession from 29 different gels and compared the mean and median of Telometric with the mean of TeloTool (Figure 3A and B, Supplementary Table S1 and see Supplementary Data S2 for information on TRF analysis and plant lines used). When probe intensity profiles are normally distributed, mean and median values are the same by definition. Telometric calculates the mean under the assumption of a normally distributed intensity profile; therefore, the median values deviate significantly for asymmetric raw data. TeloTool fits a Gaussian curve to the raw data and models a new curve based on the correction of the higher molecular weight flank. In this case, because of the symmetric curve, mean and median values will always be identical. Therefore, the tested data set consists of raw data and probe intensity corrected data. Telometric’s mean and median estimation of the raw data indicates a large overestimation of telomere length along with an increased variance from gel to gel (Figure 3A and B). The differences are highly significant (TT mean – TM mean: *P* = 9.99E^−^^11^; TT mean – TM median: *P* = 0.0013, KS-test, two-sided, α = 0.05). Furthermore, we found that TeloTool computes an average mean telomere length for the corrected data set of 3236 nt (95% CI = 3172–3301 nt), whereas Telometric’s average mean is significantly different with 4101 nt (95% CI = 3838–4363 nt) (*P* = 2.90E^−^^07^, KS-test, two-sided, α = 0.05). However, Telometric’s average median is not significantly different from TeloTool’s mean (*P* = 0.8107); this effect arises from Telometric’s inaccurate probe correction formula, which compensates the error. Averaging data over the whole lane overestimates the telomere length of the raw data, but at the same time Telometric overcorrects for probe intensity. Because the tested Col-0 accession only has a small telomere length of ∼3200 nt, the effect is not visible.

**Figure 4.**
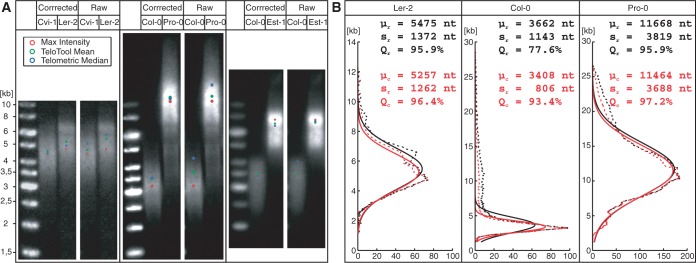
Visualization of measurements from TeloTool and Telometric on TRF smears (**A**) Comparison of the maximum intensity, TeloTool mean and Telometric median in representative Southern blots of the six tested accessions (Col-0, Pro-0, Est-1, Cvi-1, Ler-2). Telometric’s calculations lead to greater median values for the uncorrected (raw) data and smaller values for the probe-corrected data when compared with the respective mean estimated by TeloTool. The quantification of this effect can be found in Figure 3C. The colored dots represent respective mean or median parameters calculated by the two programs. (**B**) The intensity profiles with the fitted Gaussian curves for the Ler-2, Col-0 and Pro-0 lanes in A are displayed. The color-coded parameters describe the respective Gaussian curves. TeloTool’s probe correction leads to the production of distribution data that can be better described by a Gaussian function, i.e. the fit quality of the uncorrected data (Q_r_) compared with the corrected one (Q_c_) increases. kb—kilobases; µ_r_ and µ_c_—mean length of the telomere for the corrected (c) and uncorrected (r) data set. s_r_ and s_c_—standard deviation of telomere length; dashed black line—raw data; solid black line—Gaussian curve fitted to raw data; dashed red line—probe intensity corrected data; solid black line—Gaussian curve fitted to corrected data.

We further used both programs to analyze four additional *Arabidopsis* accessions, which harbor longer telomeres (Pro-0, Ler-2, Est-1, Cvi-1). The effects of incorrect probe correction are expected to affect measurements from samples exhibiting longer telomeres because of an overcompensation of the probe binding capacity. The effects of probe correction can be easily monitored by plotting the corrected versus the uncorrected data tupels (Figure 3C). Especially longer telomeres tend to be overcorrected by Telometric, e.g. a 12-kb telomere derived from the raw data is corrected to only 10 kb. The standard deviation between corrected and original data obtained with TeloTool is relatively small, which justifies the robustness of the correction. Also, the correction by TeloTool is independent of the molecular weight, which arises from the mathematical modeling approach of the intensity profile, this is, thereby, an improved method of correcting for probe intensity rather than normalizing with molecular weight. A visual representation of the tested TRF data can be found in Figure 4A. It is clearly visible that results obtained from Telometric’s analysis differ from TeloTool’s calculations. The corresponding intensity profiles for the raw and the corrected data (Figure 4B) illustrate the increased analysis power of TeloTool’s Gauss fitting approach. Through using the rising flank of the intensity profile to model the falling flank, the statistical analysis is robust and less error prone to biases.

**Figure 5.**
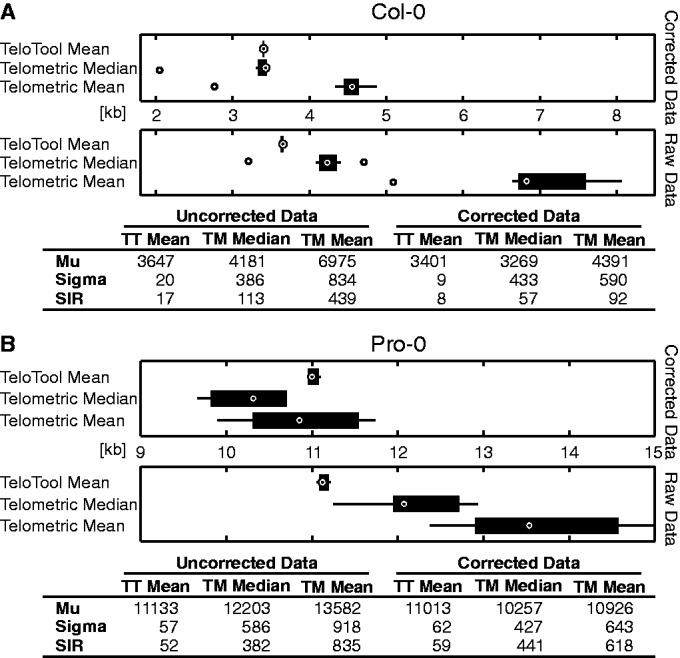
Reproducibility of measurements from TeloTool. The same gel with a TRF for Col-0 (**A**) and Pro-0 (**B**) was analyzed 10 times with both programs and the resulting mean telomere lengths were plotted as a box plot. Measurements from TeloTool were found to be highly reproducible, whereas mean and median results calculated by Telometric varied by as much as 2 kb. TM, Telometric; TT, TeloTool, mu, mean of all the mean telomere lengths, sigma, standard deviation; SIR, semi-interquartile range.

In addition to examining the intergel variation, multiple sequential analysis of the same gel was performed to address whether TeloTool can continuously reproduce the same measurements. To do this, the same gel image containing Col-0 and Pro-0 samples was analyzed 10 times with both programs. Box plots containing raw and corrected measurements were compared for each sample, measurements were found to fluctuate with Telometric, whereas measurements with TeloTool were highly reproducible (Figure 5).

## DISCUSSION

The TRF technique is a biochemical assay yielding information on the *in vivo* distribution of telomere length within a whole organism, tissue or culture. We developed a system for accurate analysis of TRF data; this method uses mathematical modeling of smear distribution to calculate telomere length data and is independent of the manual definition of lane and ladder bands particularly removing steps prone to estimator biases. Because TeloTool is based on fitting a Gaussian function through the intensity profile of the individual lanes of the gel, it is possible to yield important statistical parameters such as the median/mean, the standard deviation of the distribution and a parameter describing the fit quality. The latter depicts how well the fit describes the original data. Filtering the resulting data set with a threshold for the quality parameter greatly increases the chance to yield a significant outcome of subsequent analysis steps, as poor-quality TRF smears are excluded. These could be produced due to the use of low-grade DNA or other technical problems, which lead to a deviation in the normal distribution of the intensity profile. Typically for most bioassays, a variety of experimental factors (e.g. restriction enzymes, hybridization probes or gel quality) introduces an inter-individual variation or variation from laboratory to laboratory. To recognize such anomalies usually requires high technical expertise and a well-trained ‘judgment by eye’. Having an automatically generated and non-subjective quality parameter renders the analysis more objective. Nonetheless, the fit quality parameter is context-dependent, as it may also be used as an indicator for a non-normal telomere distribution within an organism. For example, some crosses between *Arabidopsis* accessions exhibiting different telomere lengths produce offspring with bimodal or discrete distributions (36). Therefore, this quality parameter introduces a method to identify technical problems, along with non-typical telomere distributions within an organism. Although discrete telomere distributions can easily be identified with TeloTool, further deconvolution of specific telomeric subpopulations was not implemented. The user is, nonetheless, able to use this qualitative parameter as an input for subsequent analysis steps. The quantitative parameters (central tendencies as well as dispersion values) gained from analysis of normally distributed smears may be used for more statistically complex approaches [e.g. study of mutants that display heterogeneous telomere length profiles (2,37)]. In addition, TeloTool models probe binding and produces corrected results that take into account higher signal intensities at longer telomeres. With this modeling approach, TeloTool does not only correct for probe intensity but also for inhomogeneity in the electrical field during the gel run and gel impurities. Using this approach, our data show TeloTool can consistently measure telomere length over many gels giving little deviation (Figure 3A and B) along with consistent measurements of the same sample from the same gel (Figure 5) revealing high reproducibility of the results.

As mentioned previously, many methods have been described to extract telomere length from raw TRF data. As described at the start of the article, averaging probe intensity over the whole lane was previously used to quantify average telomere length; this method, however, introduces severe biases as shown within this article. Other methods were also used in a number of studies, this includes manual definition of an analysis window, i.e. to only use certain parts of the TRF smear for analysis. This practice is only rarely allowed and leads to largely biased results if applied incorrectly. Only a few studies report to have used an analysis window that is appropriate to the investigated organism (17,38). In another case, a manually defined analysis window was selected to cover the majority of the probe distribution (39). This procedure is problematic as the signal intensity is often dependent on exposure time. The next attempt was to analyze the telomere length from the bottom of the smear to just below the limit of mobility (40). This approach also leads to biases, as it is hard to experimentally determine the limit of mobility and, furthermore, it is also highly variable between individuals of the same species (41). Another study focuses on the shortest telomeres with statistical identification of the analysis window by searching for the highest correlation between parameters of interest (42). However, this approach assumes that there is actually a correlation (e.g. telomere length and age) and, because such studies want to show that there is a correlation in the first place, vastly biased. Because TeloTool fits a Gaussian curve through the whole TRF smear, biases introduced by incorrectly chosen analysis windows are removed.

On the one hand, we have shown that Telometric overestimates telomere length derived from the raw data in comparison with TeloTool’s results. On the other hand, when plotting measurements of probe-intensity-corrected data for both programs, Telometric was shown to overcorrect longer telomeres, e.g. a 12-kb telomere would be as much as 2 kb shorter (Figure 3C). Telometric’s probe correction represents a simple normalization by molecular weight and, therefore, assumes that first, every hypothetical binding site is actually bound and second, the labeling of every bound probe is intact. In reality, it is more likely that probes bind in a probabilistic way and some of them may have lost their label. Therefore, it is most likely that the number of probes binding to the telomeric DNA as calculated by Telometric is vastly overestimated leading to a large overcorrection at longer telomeres. TeloTool’s probe correction on the other hand is based on the mathematic modeling of the raw data distribution not on the assumption that a specific number of probes bind. Using the best Gaussian fit through the raw data, the deviation from a normal distribution is calculated and eventually the raw data is corrected with precisely this error creating the corrected data set. This procedure is more flexible, as it takes multiple biases into account such as probe intensity, gel impurities, inhomogeneity within the electrical field and local background heterogeneity. The latter would induce fluctuations in the intensity profile, which has a large impact on the segmental Telometric analysis method, but are completely irrelevant for the analysis with TeloTool.

TeloTool is able to analyze data that have previously shown to be difficult to quantify. Various studies in *Arabidopsis* have revealed a number of mutants that display telomere defects and, therefore, skewed or asymmetric telomere profiles. *Arabidopsis stn1* and *ctc1* mutants have both shown to contain extensive telomere length heterogeneity in comparison with wild-type plants (2,37). Using TeloTool, it would be possible to compare dispersion information between wild-type and mutant samples. In addition, it may also be possible to make comparisons between multiple combinations of mutants that display heterogeneous telomere profiles. Crossing in additional mutants may exacerbate this phenotype; comparison between diffuse TRF smears could be difficult by eye and easy to quantify using TeloTool. Drastic length fluctuations are also seen within a number of studies investigating mutants with disrupted telomeres; *Arabidopsis Ku70* and *Ku80* mutants have previously shown to display extensive telomere length elongation (43,44). Analysis of such data with TeloTool allows exact quantification so that appropriate significance tests can be performed. Length variation in natural populations of *Arabidopsis* has also previously been recorded (5). This variation has also been experienced in yeast, humans, mice and maize and has previously been used for mapping-based approaches (4,45–51). Increased accuracy of telomere length measurements may improve the significance of quantitative trait loci mapping data or uncover new peaks for those studies that have used TRFs to quantify telomere length. TRFs are also frequently used to profile telomere length in a number of studies investigating human disease and cellular lifespan [see Supplementary Figure S1, e.g. of TRF analysis from HeLa cells; the gel was taken from a previous publication (52)]. For example, TRF data were used to show that telomerase-positive human cells display increased telomere length and an elongated life span (53). In addition, Agarwal *et al.* (2010) showed that human fibroblasts with mutations in dyskerin (DKC1), a known cause of dyskeratosis congenita (DKC), displayed induction of endogenous telomerase activity and an eventual increase in telomere length (54). Such studies would benefit from the increased accuracy introduced through TeloTool-based measurements.

Using TeloTool, it is now possible to accurately extract telomere length and heterogeneity information from Southern blot data to analyze TRF smears. This approach is applicable to any TRF-based study from any organism and provides a quick and reproducible method for telomere length analysis.

## SUPPLEMENTARY DATA

Supplementary Data are available at NAR Online.

## FUNDING

Austrian Science Fund (FWF), [Y418-B03] from Karel Riha and the Austrian Science Fund (FWF), [DK W1207] and the EU FP6 NoE (EURASNET) [LSHG-CT-2005-518238] from Andrea Barta. Furthermore, the work described in this article was done within the FIT-IT project number 835918 ‘NanoDetect: A Bioinformatics Image Processing Framework for Automated Analysis of Cellular Macro and Nano Structures’ sponsored by the Austrian Research Promotion Agency (FFG). Funding for open access charge: Austrian Science Fund (FWF), [Y418-B03] from Karel Riha.

*Conflict of interest statement*. None declared.

## Supplementary Material

Supplementary Data
