# Supplementary material for: TeloTool: a new tool for telomere length measurement from terminal restriction fragment analysis with improved probe intensity correction
Source: Nucleic Acids Res. 2013 Dec 22;42(3):e21. doi: 10.1093/nar/gkt1315 (PMC3919618; doi:10.1093/nar/gkt1315)
Supplement: Supplementary Data [file supp_42_3_e21__index.html]

TeloTool: a new tool for telomere length measurement from terminal restriction fragment analysis with improved probe intensity correction — TeloTool: a new tool for telomere length measurement from terminal restriction fragment analysis with improved probe intensity correction — TeloTool: a new tool for telomere length measurement from terminal restriction fragment analysis with improved probe intensity correction — Supplementary Data 

# TeloTool: a new tool for telomere length measurement from terminal restriction fragment analysis with improved probe intensity correction

## Supplementary Data

files

**Files in this Data Supplement:**

- Supplementary Data - pdf file
- Supplementary Data - docx file
- Supplementary Data - docx file
- Supplementary Data - xlsx file
