# Supplementary material for: TeloTool: a new tool for telomere length measurement from terminal restriction fragment analysis with improved probe intensity correction
Source: Nucleic Acids Res. 2013 Dec 22;42(3):e21. doi: 10.1093/nar/gkt1315 (PMC3919618; doi:10.1093/nar/gkt1315)
Supplement: Supplementary Data [file supp_gkt1315_nar-02996-met-g-2013-File007.pdf]

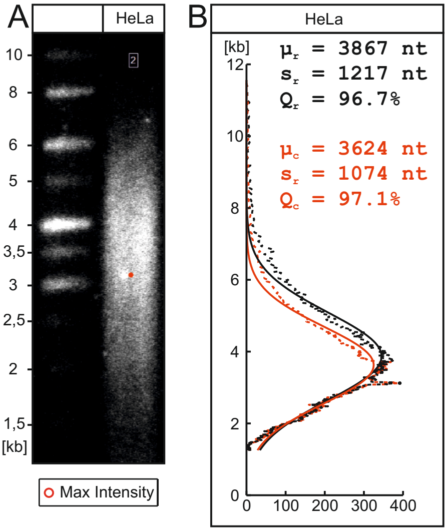

Supp. Fig. S1 **Telomere measurement from HeLa cells using TeloTool.** To show TeloTool based measurements from human cell culture, we used previously published HeLa denaturing gels from Kazda *et al.* (52). A) Image representing telomeric smear and maximum intensity of the raw probe distribution. B) Raw (black) and corrected (red) probe intensity distributions. Raw probe intensities are represented by dashed lines, solid lines show the respective Gaussian fit. μ_r & μ_c – mean length of the telomere for the corrected(c) and uncorrected (r) data set; s_r & s_c – standard deviation of telomere length; Q_r & Q_c – fit quality to of curve to raw data.
