# Supplementary material for: TeloTool: a new tool for telomere length measurement from terminal restriction fragment analysis with improved probe intensity correction
Source: Nucleic Acids Res. 2013 Dec 22;42(3):e21. doi: 10.1093/nar/gkt1315 (PMC3919618; doi:10.1093/nar/gkt1315)
Supplement: Supplementary Data [file supp_gkt1315_nar-02996-met-g-2013-File008.docx]

**Theoretical considerations - Nonlinearity effects in hybridization kinetics**

Described in the manuscript is a method to correct for the increasing probe intensity at longer telomeres; this is necessary to yield accurate mean telomere length values. Former analyses approaches corrected for probe intensity by normalizing the data simply with the molecular weight assuming a linearity of probe binding i.e. that all theoretically possible binding sites are saturated with effective probes. This scenario is unlikely since many effects lead to non-linear probe binding. These include not only theories on general hybridization kinetics and labeling stoichiometry, but also the accessibility of the probe binding sites can be disturbed (metastable secondary structures of the target, effects caused by the 3D matrix the telomeric DNA is bound to). We will elaborate on them in the following theoretical considerations. The dynamics of non-linear probe binding are well documented and we, therefore, believe that the rising flank (low-molecular weight telomeres) of the distribution can be used for the correction of the falling flank.

In our considerations about the influence of telomere length on binding kinetics, a few assumptions were made. First we assumed a pseudo first order oligonucleotide binding kinetic model, which means a strong overshoot of probe molecules relative to telomeric DNA during the hybridization process. Secondly, we interpret the porous matrix of the membrane as a 3D substrate, which induces 3D sample diffusion. Moreover, we also assumed that no other competitors (even unspecific) are present in hybridization solutions.

***General hybridization kinetics (special case of targets carrying repeats)***

To study the length dependent kinetics of telomeres with long repetitive sequences first we would like to address the mechanics behind simple oligonucleotide binding kinetics in a 3D volume ([1](#_ENREF_1)).

According to the equations presented in the review on DNA binding kinetics by Wetmur et al. the following dependence of the binding rate constant can be derived.

| $k_{2}=\frac{k_{N}^{'}\sqrt{L_{s}}}{N},$ | (Eq.1) |
| --- | --- |

where $k_{2}$ is the rate constant ($M^{-1}s^{-1}$),

$L_{s}$ is the length of the shortest strand participating in duplex formation,

$N$ is the complexity or the total number of base pairs present in non-repeating sequence,

$k_{N}^{'}$is the nucleation rate constant (strongly dependent on ionic strength, temperature and viscosity).

The inverse dependence of $k_{2}$ on $N$ results from mass action, where at constant $C_{o}$ (molar nucleotide residue concentration), increasing $N$ means a lower concentration of any particular sequence ([1](#_ENREF_1)). The $\sqrt{L_{s}}$ dependence implies that fewer binding partners of a complementary sequence are available for reaction when the sequence gets longer. For self-complimentary interactions (secondary structure of the target), $N$ is equal $L_{s}$ (holds true also for short telomeres or telomere repeats). Consequently, for short hybridizing sequences, or hybridization of targets with many repeats (like telomere sequences), the kinetic depends mostly on $k_{N}^{'}$. In this case, the $k_{N}^{'}$ for self-association (or hybridization to short repeats) is much faster than for regular DNAs where the possibility of nucleation decreases with length ($k_{N}^{'}=k_{2}\sqrt{L_{s}}$). The effects of solvent viscosity (molecular diffusion), influencing $k_{N}^{'}$ and consequently DNA hybridization will be described later ([1](#_ENREF_1)).

These considerations clearly show that in the case of telomeric sequences, due to the linearity of the rate constant dependence, the nonlinearities in hybridization behavior at long telomeres cannot be explained by simple binding kinetics. The nonlinear behavior of binding kinetics results from an interplay of several biochemical and biophysical processes which need to be included in our considerations.

***Labeling stoichiometry of the probe:***

One reason for signal nonlinearities is the loss of the _32_P labels of the probe oligonucleotide. Therefore, the signal scales no longer linear with the number of telomere repeats and displays a lower overall signal for long telomeres.

***Anomaly in kinetics on solid supports:***

In the past, it has been reported that 3D micro-porous surfaces (e.g. nitrocellulose) have a greater number of potential immobilization sites than, for example, 2D modified glass ([2](#_ENREF_2)). Higher local densities of DNA have an effect on two parameters, namely diffusion and secondary DNA structures, which are crucial for the hybridization kinetics. Hybridization kinetics on high-capacity supports are more complex than with flat glass because mass transfer and non-uniformity within the film must be considered ([3](#_ENREF_3)). Although a variety of high-capacity platforms have been investigated experimentally, theoretical modeling and investigation of the fundamental parameters governing the performance has received less attention in the past. Livshits and Mirzabekov proposed and tested the theory of “retardation” ([3](#_ENREF_3),[4](#_ENREF_4)).

***Diffusion of long telomeres in porous supports:***

Hybridization studies on 3D nitrocellulose membranes in Southern blots showed that increasing the molecular weight of the target DNA transferred to the matrix leads to a decrease in the rate of hybridization ([5](#_ENREF_5)). These nonlinearities were brought into relation with the size dependent changes in the molecular diffusion of the transferred DNA (“washed out” targets) on the membrane during incubation with the radioactive probe (even if sample shaking has been introduced). Due to an adhesive interaction of the DNA with the membrane, the assumption of an immobilized, transferred DNA is not realistic. Theoretical explanation for this phenomenon performed by Van Oss *et al.* show that the interaction of DNA with a porous matrix depends on DNA length ([6](#_ENREF_6)).

***Complex hybridization kinetics due to probe diffusion in porous supports:***

The theory proposes that the diffusion of probes into the matrix during hybridization is controlled by the same type of interactions as the target DNA. Moreover, within a polymer gel, the probes are impeded by repeated association and dissociation with the “washed out” target DNA in the porous membrane. For example, when testing hybridization in flow chambers (mixing environment) using short 20nt oligonucleotides, the diffusion coefficient has been estimated by Glazer *et al.* ([3](#_ENREF_3)).

***DNA repeats (Telomeres) form self-complementary metastable states:***

Beside the environmental parameters such as matrix and diffusion, we as well have to consider the structural properties of telomeric DNA due to its repetitive sequence. Regarding ([7](#_ENREF_7)), longer telomeric structures with repeatable sequences can form metastable intermediate states (secondary DNA structures), which impact probe hybridization kinetics ([7](#_ENREF_7),[8](#_ENREF_8)). At low concentrations, the time spent in metastable intermediates is negligible compared to the typical time between attachment events. For high local concentrations (like in a porous substrate), metastable states influence probe binding rate constants ([7](#_ENREF_7)) having an impact on the association and dissociation of the probes and DNA. The accurate measurement of the transition rates between all registers is impractical due to the enormous number of possibilities and the large range of transition rates ([7](#_ENREF_7)).

***References***:

1. Wetmur, J.G. (1991) DNA probes: applications of the principles of nucleic acid hybridization. *Crit. Rev. Biochem. Mol. Biol.*, **26**, 227-259.

2. Stillman, B.A. and Tonkinson, J.L. (2001) Expression microarray hybridization kinetics depend on length of the immobilized DNA but are independent of immobilization substrate. *Anal. Biochem.*, **295**, 149-157.

3. Glazer, M.I., Fidanza, J.A., McGall, G.H., Trulson, M.O., Forman, J.E. and Frank, C.W. (2007) Kinetics of oligonucleotide hybridization to DNA probe arrays on high-capacity porous silica substrates. *Biophys. J.*, **93**, 1661-1676.

4. Livshits, M.A. and Mirzabekov, A.D. (1996) Theoretical analysis of the kinetics of DNA hybridization with gel-immobilized oligonucleotides. *Biophys. J.*, **71**, 2795-2801.

5. Flavell, R.A., Birfelder, E.J., Sanders, J.P. and Borst, P. (1974) DNA-DNA hybridization on nitrocellulose filters. 1. General considerations and non-ideal kinetics. *Eur. J. Biochem.*, **47**, 535-543.

6. Van Oss, C.J., Good, R.J. and Chaudhury, M.K. (1987) Mechanism of DNA (Southern) and protein (Western) blotting on cellulose nitrate and other membranes. *J Chromatogr*, **391**, 53-65.

7. Ouldridge, T.E., Sulc, P., Romano, F., Doye, J.P. and Louis, A.A. (2013) DNA hybridization kinetics: zippering, internal displacement and sequence dependence. *Nucleic Acids Res.*, **41**, 8886-8895.

8. Gao, Y., Wolf, L.K. and Georgiadis, R.M. (2006) Secondary structure effects on DNA hybridization kinetics: a solution versus surface comparison. *Nucleic Acids Res.*, **34**, 3370-3377.
