# Supplementary material for: TeloTool: a new tool for telomere length measurement from terminal restriction fragment analysis with improved probe intensity correction
Source: Nucleic Acids Res. 2013 Dec 22;42(3):e21. doi: 10.1093/nar/gkt1315 (PMC3919618; doi:10.1093/nar/gkt1315)
Supplement: Supplementary Data [file supp_gkt1315_nar-02996-met-g-2013-File009.docx]

**Plant Material**

*Arabidopsis* lines were ordered from the European Arabidopsis stock centre (NASC): Col-0 (N22625), Cvi-1 (N8580), Est-1 (N22629), Ler-2 (N8581), Pro-0 (N22649). Plants were grown for 27 days in greenhouse conditions (22^o^C, 16hr/8hr Light/Dark period).

**DNA extraction**

Tissue from 5 plants was ground in liquid nitrogen and transferred to 4ml CTAB DNA extraction buffer (1.4M NaCl, 20g/L CTAB (hexadecyltrimethylammonium bromide (Sigma)), 0.1M Tris-HCl pH8). After incubation at 65^o^C for 1hr, DNA was extracted with an equal volume of Phenol:Chloroform:Isoamyl alcohol 25:24:1 (Sigma) and precipitated with Isopropanol. The resulting pellet was resuspended in dH_2_O and subject to RNAse treatment (10mg/ml RNAse A).

**TRF analysis**

TRFs were performed using Tru1I (Thermo Fisher Scientific) and _32_P 5’ end-labelled T_3_AG_3_ oligonucleotide probes according to Fitzgerald et al. 1999.
